# Clinico-aetiological profile, risk factors for decompensation and outcomes in paediatric chronic liver disease: a retrospective cohort study from a tertiary care centre in South India

**DOI:** 10.1186/s12887-026-07499-7

**Published:** 2026-08-10

**Authors:** Sarthak Katyal, Karen Janice Moras, Suneel Mundkur, Shrikiran A Hebbar, Pushpa Kini, Ramesh Bhat, Leslie Edward S Lewis, Kaushik H

**Affiliations:** Department of Paediatrics, Kasturba Medical College, Manipal Academy of Higher Education, Manipal, Karnataka 576104 India

**Keywords:** Malnutrition, Decompensation, Heaptic Encephalopathy, Hyponatraemia, Gastrointestinal bleeding

## Abstract

**Background:**

Chronic liver disease (CLD) in children encompasses a heterogeneous group of disorders with aetiologies that differ significantly from adults. Data from low- and middle-income countries, particularly India, remain limited, especially regarding determinants of decompensation and outcomes.

**Methods:**

A retrospective observational study was conducted at a tertiary care centre in South India including children aged 3 months to 18 years admitted with CLD between January 2017 and December 2022. Clinical presentation, nutritional status, aetiology, decompensation profile, investigations, complications and outcomes were analysed. Decompensation was defined as the presence of ascites, hepatic encephalopathy or gastrointestinal bleeding. Statistical analysis was performed using SPSS v21.0; chi-square and Fisher’s exact tests were applied for categorical comparisons, with *p* < 0.05 considered significant.

**Results:**

Ninety-eight children were included with equal gender distribution. Wilson disease (36.7%) and extrahepatic biliary atresia (EHBA, 28.6%) were the leading aetiologies. Decompensation occurred in 80.6% of children; hyponatraemia (36.7%), sepsis (35.4%), gastrointestinal bleeding (34.2%) and pneumonia (31.6%) were the most frequent precipitants. Malnutrition was present in 62.2% of patients. Overall mortality was 20.4%. Aetiology-specific mortality differed significantly (χ²=12.47, *p* = 0.014), being highest in EHBA (35.7%). On univariable analysis, recurrent decompensation (≥ 2 episodes) was associated with higher mortality (*p* = 0.032).

**Conclusion:**

Paediatric CLD presents with advanced disease and a high burden of decompensation in this setting. Early diagnosis, nutritional optimisation and prompt management of infections and electrolyte disturbances are essential to improve outcomes. Aetiology-specific surveillance and structured decompensation protocols should be prioritised.

**Graphical abstract:**

**Figure:**
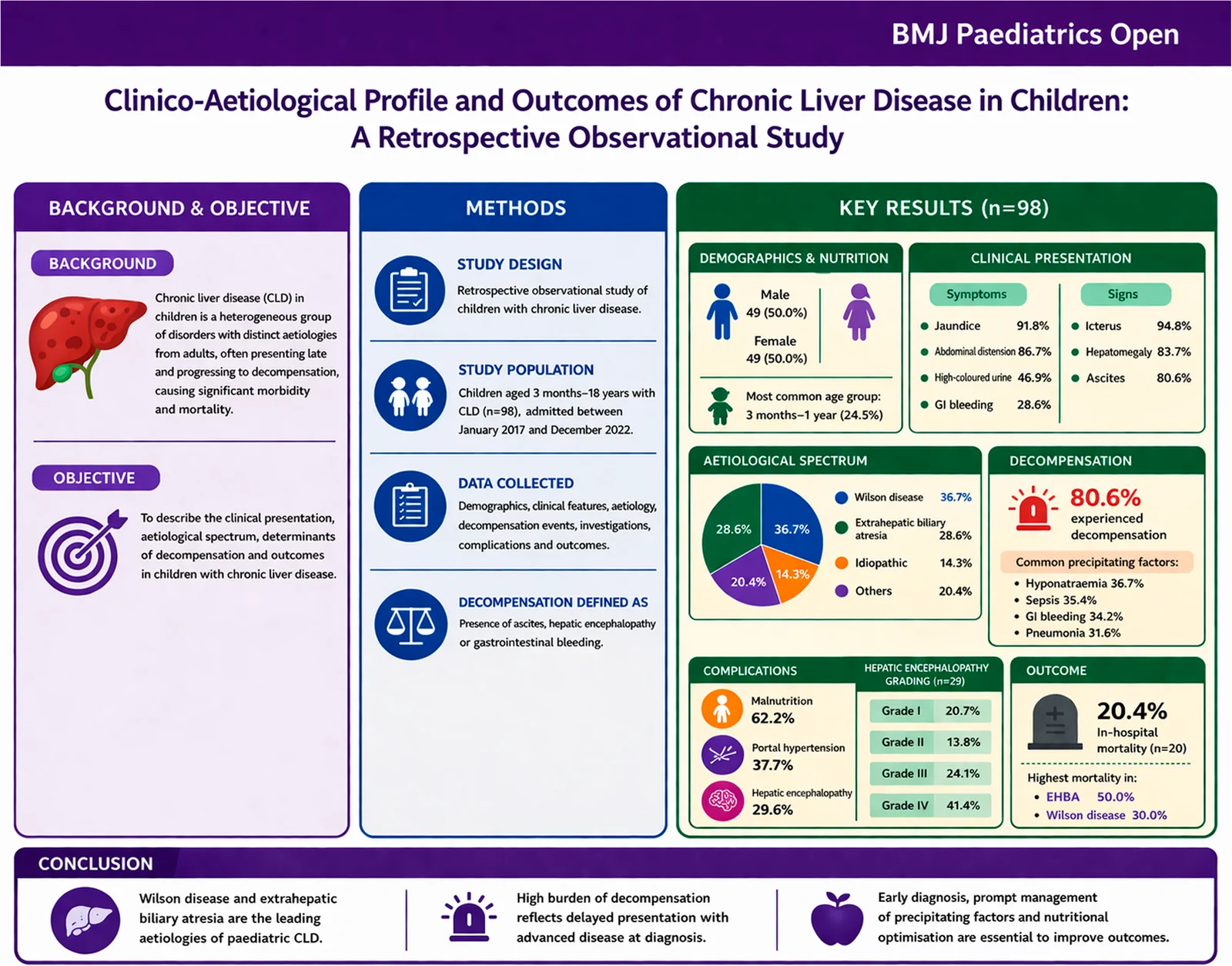

**Supplementary Information:**

The online version contains supplementary material available at https://doi.org/10.1186/s12887-026-07499-7.

## Key message

What is already known on this topic: Chronic liver disease in children is predominantly caused by metabolic, genetic and developmental conditions. The disease course involves progression to decompensation, which is associated with substantially higher mortality. Published data from India are limited, particularly regarding precipitants of decompensation and predictors of outcomes.

What this study adds: This study demonstrates that 80.6% of paediatric CLD patients presented in a decompensated state, with Wilson disease (36.7%) and EHBA (28.6%) as the leading aetiologies. Malnutrition, hyponatraemia, gastrointestinal bleeding, pneumonia and hepatic encephalopathy were identified as factors significantly associated with decompensation on univariable analysis. Recurrent decompensation (≥2 episodes) was associated with nearly four-fold higher mortality odds on univariable analysis (OR 3.84, 95% CI 1.12–13.14).

How this study might affect research, practice or policy: These findings underscore the need for early diagnosis, structured nutritional assessment, aggressive electrolyte monitoring and infection surveillance in paediatric CLD management. Aetiology-specific surveillance programmes and standardised decompensation protocols are needed in tertiary care settings in low- and middle-income countries.

**Supplementary Information:**

The online version contains supplementary material available at https://doi.org/10.1186/s12887-026-07499-7.

## Introduction

Chronic liver disease (CLD) in children represents a diverse group of progressive hepatic disorders characterised by persistent inflammation, fibrosis and eventual cirrhosis [1]. Unlike adults, where CLD is largely driven by lifestyle and viral factors, paediatric CLD is predominantly caused by metabolic, genetic and developmental conditions, resulting in a distinct clinical and aetiological spectrum [2]. Cirrhosis represents the end stage of chronic hepatic injury and is associated with architectural distortion of the liver, portal hypertension and progressive hepatic insufficiency [1]. The burden of paediatric CLD has been increasing globally, contributing significantly to morbidity, healthcare utilisation and mortality, particularly in low- and middle-income countries where delayed diagnosis and limited access to specialised care are common [3, 4]. 

The natural course of CLD involves progression from a compensated phase to decompensation, characterised by the development of ascites, hepatic encephalopathy or gastrointestinal bleeding [5]. Decompensation marks a critical turning point in disease trajectory and is associated with a substantial increase in mortality risk. Identification of precipitating factors such as infections, electrolyte disturbances and gastrointestinal bleeding is therefore essential for timely intervention. The aetiological spectrum of paediatric CLD varies with age: biliary atresia is the leading cause in infancy, whereas metabolic disorders such as Wilson disease and autoimmune hepatitis predominate in older children [2, 6]. 

There is a relative paucity of comprehensive data from India describing the clinico-aetiological profile, risk factors for decompensation and outcomes in paediatric CLD. Existing studies are often limited by small sample sizes or focus on specific disease subgroups [2, 6]. This study aims to evaluate the clinical presentation, aetiological spectrum, determinants of decompensation and outcomes in children with CLD presenting to a tertiary care centre in South India, and to identify statistically significant associations with decompensation and mortality.

## Methods

This retrospective observational study was conducted in the Department of Paediatrics at Kasturba Hospital, Manipal, a tertiary care teaching centre in South India. The study included all children aged between 3 months and 18 years admitted with a diagnosis of CLD between January 2017 and December 2022. Chronic liver disease was defined as evidence of persistent hepatic dysfunction lasting more than three months, or clinical, biochemical and radiological features suggestive of chronic liver injury (hepatomegaly, splenomegaly, portal hypertension or cirrhosis). Decompensation was defined as the presence of ascites, hepatic encephalopathy or gastrointestinal bleeding. The primary outcome was in-hospital mortality; secondary outcomes included occurrence of decompensation and its precipitating factors. Children younger than 3 months, those diagnosed with hepatocellular carcinoma and those with congenital haemolytic anaemias were excluded. Nutritional status was evaluated using the Indian Academy of Pediatrics (IAP) classification for children below 5 years and WHO BMI criteria for those aged 5 years and above.

Data were analysed using SPSS version 21.0. Continuous variables were expressed as mean ± standard deviation; categorical variables as frequencies and percentages. Chi-square and Fisher’s exact tests were used for comparisons of categorical variables. A p-value less than 0.05 was considered statistically significant. Factors associated with decompensation were examined using univariable analysis. Odds ratios (OR) with 95% confidence intervals (CI) were calculated for variables achieving significance on univariable testing. Recurrent decompensation (≥ 2 episodes) was analysed as a factor associated with mortality rather than with decompensation, because recurrence is by definition confined to decompensated patients and a decompensated-versus-non-decompensated comparison would be tautological. As this was a retrospective observational study, all eligible patients meeting the inclusion criteria during the study period were included, and no formal sample size calculation was performed. Given the relatively small sample size and number of outcome events, multivariable analysis was not undertaken to avoid model overfitting and unstable estimates; therefore, all reported associations are univariable and should not be interpreted as independent of other factors. International normalised ratio (INR) and prothrombin time were not uniformly recorded across the retrospective record set; formal classification of acute liver failure (ALF) or acute-on-chronic liver failure (ACLF) using standardised coagulopathy thresholds was therefore not undertaken. This study is also subject to potential selection and referral bias, as it was conducted in a tertiary care centre where patients are more likely to present with advanced disease, which may overestimate the burden of decompensation and associated complications. Ethical approval was obtained from the Institutional Ethics Committee of Kasturba Medical College and Kasturba Hospital, Manipal Academy of Higher Education (IEC-220/2023); individual consent was waived given the retrospective anonymised design.

## Results

**Fig. 1:**
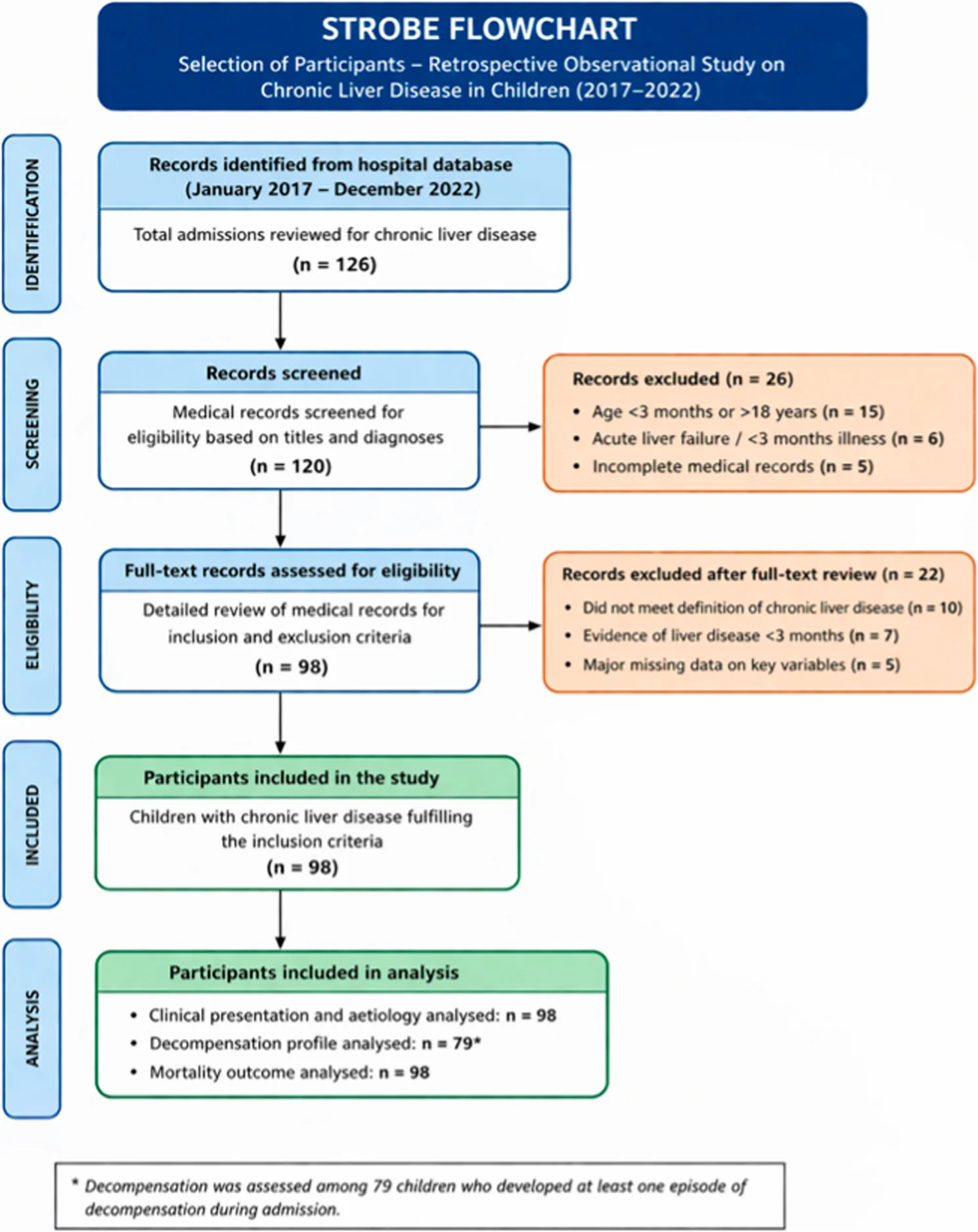
STROBE flow diagram of participant selection. Flow diagram showing identification, screening, eligibility, and inclusion of children with chronic liver disease between January 2017 and December 2022. Of 126 records identified, 98 met inclusion criteria and were analysed. Decompensation analysis was performed in 79 patients

**Fig. 2:**
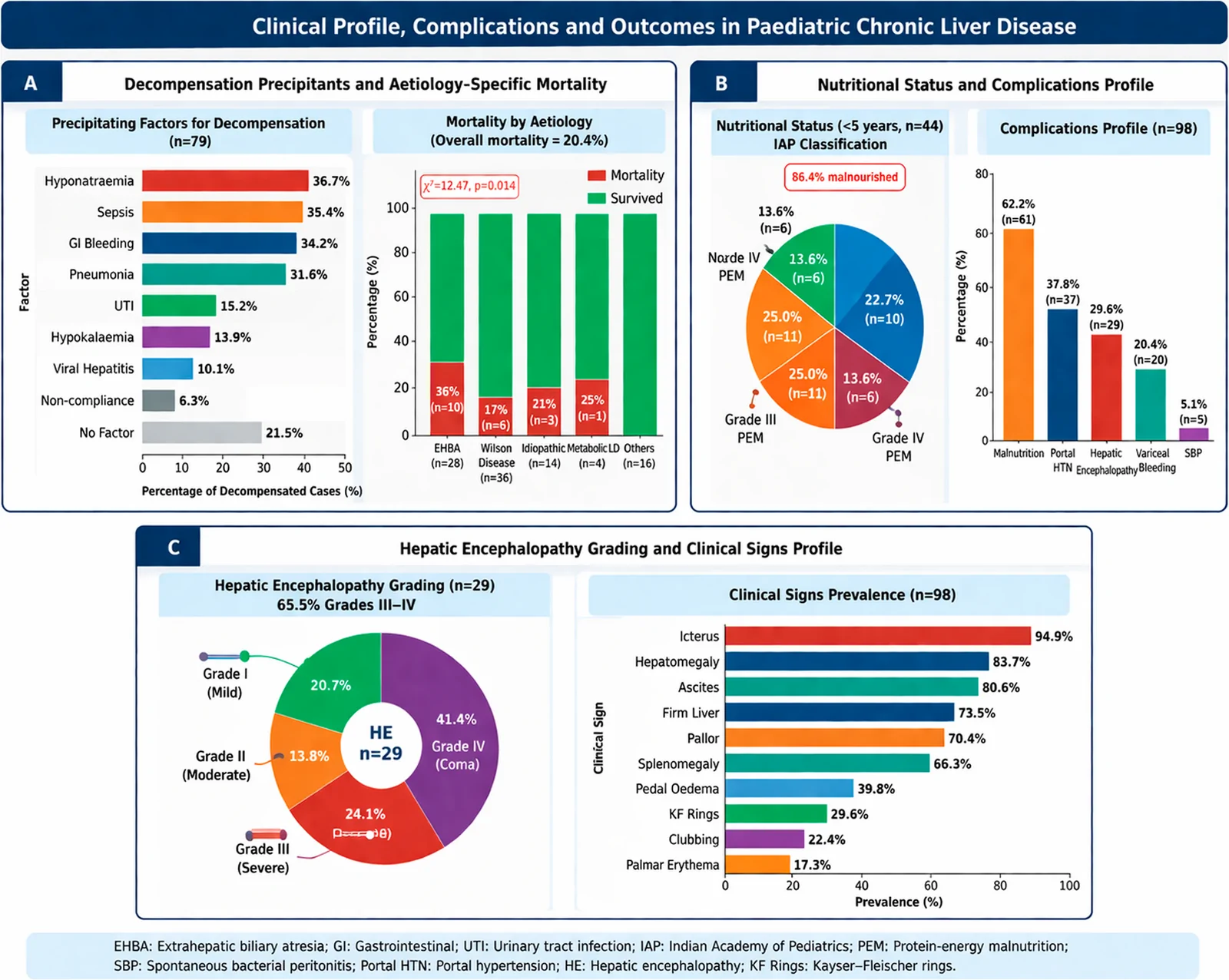
Clinical profile, complications and outcomes in paediatric chronic liver disease. **A** Precipitating factors for decompensation and aetiology-specific mortality. **B** Nutritional status in children aged < 5 years and overall complications profile. **C** Hepatic encephalopathy grading and prevalence of clinical signs. Decompensation was assessed in 79 patients. Overall mortality was 20.4%, with aetiology-specific differences (χ²=12.47, *p* = 0.014). EHBA had the highest case-fatality rate

A total of 98 children with CLD were included, with equal gender distribution (49 males and 49 females) (Table 1; Fig. 1). The largest age group was 3 months to 1 year (24.5%) (Table 1). Wilson disease was the most common aetiology (36.7%), followed by EHBA (28.6%), idiopathic CLD (14.3%), Budd–Chiari syndrome (7.1%), autoimmune hepatitis (5.1%) and metabolic liver disease (4.1%) (Table 1). Malnutrition was highly prevalent: among children aged < 5 years (*n* = 44), 86.4% had protein–energy malnutrition (PEM), with Grade II and III each accounting for 25.0% (Table 1; Fig. 2B). Among those aged ≥ 5 years (*n* = 54), 42.6% were underweight by WHO BMI criteria (Table 1).

**Table 1:** Demographic characteristics and nutritional status of children with chronic liver disease (*n* = 98)

Variable	Category	*n* (%)
Gender	Male	49 (50.0)
	Female	49 (50.0)
Age group (years)	0.25–1	24 (24.5)
	1–5	20 (20.4)
	5–10	21 (21.4)
	10–15	21 (21.4)
	15–18	12 (12.2)
Aetiology of CLD	Wilson disease	36 (36.7)
	EHBA	28 (28.6)
	Idiopathic CLD	14 (14.3)
	Budd–Chiari syndrome	7 (7.1)
	Autoimmune hepatitis	5 (5.1)
	Metabolic liver disease (tyrosinaemia)	4 (4.1)
	Others*	4 (4.1)
Nutritional status < 5 yrs (*n* = 44) — IAP classification	Normal	6 (13.6)
	Grade I PEM	10 (22.7)
	Grade II PEM	11 (25.0)
	Grade III PEM	11 (25.0)
	Grade IV PEM	6 (13.6)
Nutritional status ≥ 5 yrs (*n* = 54) — WHO BMI criteria	Normal BMI	31 (57.4)
	Underweight	23 (42.6)

*PEM* Protein-energy malnutrition, *BMI* Body mass index, *IAP* Indian Academy of Pediatrics, *WHO* World Health Organization

*Others include aetiologies each occurring in fewer than four patients

The clinical profile was dominated by features of advanced liver disease. The most common symptoms were jaundice (91.8%) and abdominal distension (86.7%), followed by high-coloured urine (46.9%) and pale stools (30.6%) (Supplementary Table S1). On examination, icterus (94.8%), hepatomegaly (83.7%) and ascites (80.6%) were the predominant signs, along with splenomegaly (66.3%) and pallor (70.4%) (Supplementary Table S1; Fig. 2C). Kayser–Fleischer rings were present in 29.5% of patients (Supplementary Table S1). Decompensation was observed in 80.6% (*n* = 79) of patients (Table 2; Fig. 2A). Among all patients, 48.0% had one episode, 29.6% had two episodes and 3.0% had three episodes of decompensation (Table 2). Recurrent decompensation (≥ 2 episodes) was associated with significantly higher mortality compared with a single episode on univariable analysis (OR 3.84, 95% CI 1.12–13.14, *p* = 0.032). On univariable analysis, malnutrition (OR 3.71, *p* = 0.013), hyponatraemia (OR 4.89, *p* = 0.031), gastrointestinal bleeding (OR 9.24, *p* = 0.009), pneumonia (*p* = 0.003) and hepatic encephalopathy (*p* < 0.001) were significantly associated with decompensation (Table 3). Among decompensated patients, the most common precipitating factors were hyponatraemia (36.7%), sepsis (35.4%), gastrointestinal bleeding (34.2%) and pneumonia (31.6%) (Table 2; Fig. 2A).

**Table 2:** Univariable analysis of factors associated with decompensation (Decompensated *n* = 79 vs. Non-decompensated *n* = 19)

Variable	Decompensated (*n* = 79)	Non-decompensated (*n* = 19)	OR (95% CI)	*p*-value
Malnutrition (any grade)	54 (68.4)	7 (36.8)	3.71 (1.31–10.52)	0.013*
Hyponatraemia	29 (36.7)	2 (10.5)	4.89 (1.06–22.60)	0.031*
Sepsis	28 (35.4)	3 (15.8)	2.91 (0.77–11.01)	0.116
GI bleeding at presentation	27 (34.2)	1 (5.3)	9.24 (1.18–72.4)	0.009*
Pneumonia	25 (31.6)	0 (0.0)	N/A (Fisher)	0.003*
Wilson disease	27 (34.2)	9 (47.4)	0.57 (0.21–1.57)	0.277
EHBA	26 (32.9)	2 (10.5)	4.16 (0.89–19.46)	0.062
Age < 1 year	19 (24.1)	5 (26.3)	0.89 (0.29–2.77)	0.845
Hepatic encephalopathy	29 (36.7)	0 (0.0)	N/A (Fisher)	< 0.001*

Chi-square test used unless indicated; Fisher’s exact test applied where expected cell count < 5. The variable “recurrent episodes (≥ 2)” has been removed from this analysis because recurrence is definitionally confined to decompensated patients, rendering a decompensated-versus-non-decompensated comparison tautological; recurrent decompensation is instead reported as a factor associated with mortality (OR 3.84, 95% CI 1.12–13.14, *p* = 0.032). Aetiologies with fewer than five patients in either group (autoimmune hepatitis, Budd–Chiari syndrome, metabolic liver disease) were not tested individually owing to insufficient cell counts; only the two commonest aetiologies (Wilson disease and EHBA) were analysed

*OR* Odds ratio, *CI* Confidence interval, *GI* Gastrointestinal, *EHBA* Extrahepatic biliary atresia

*Statistically significant (*p* < 0.05)

**Table 3:** Decompensation profile and precipitating factors (*n* = 98; decompensated *n* = 79)

Variable	*n*	% (of relevant denominator)
Decompensation status (*n* = 98)		
Present	79	80.6
Absent	19	19.4
Episodes of decompensation (*n* = 98)		
0 episodes	19	19.4
1 episode	47	48.0
2 episodes	29	29.6
3 episodes	3	3.0
Precipitating factors (*n* = 79 decompensated)		
Hyponatraemia	29	36.7
Sepsis	28	35.4
Gastrointestinal bleeding	27	34.2
Pneumonia	25	31.6
No identifiable factor	17	21.5
Urinary tract infection	12	15.2
Hypokalaemia	11	13.9
Viral hepatitis	8	10.1
Non-compliance with treatment	5	6.3

Percentages for precipitating factors are of *n* = 79 decompensated patients; multiple precipitants per patient are possible

Complications included malnutrition (62.2%), portal hypertension (37.7%), hepatic encephalopathy (29.6%), variceal bleeding (20.4%) and spontaneous bacterial peritonitis (5.1%) (Supplementary Table S3; Fig. 2B). Among patients with hepatic encephalopathy (*n* = 29), 41.4% had Grade IV and 24.1% had Grade III disease (Supplementary Table S3; Fig. 2C). Blood cultures were positive in 24.1% (7/29), most commonly *Streptococcus pneumoniae* (*n* = 3), followed by *Klebsiella pneumoniae* (*n* = 2), *Pseudomonas aeruginosa* (*n* = 1) and *Escherichia coli* (*n* = 1); urine cultures were positive in 50.0% (6/12), predominantly *Escherichia coli* (*n* = 4) and *Enterobacter* species (*n* = 2) (Supplementary Table S2). Ultrasonography showed coarse hepatic echotexture in 89.8%, with portal hypertension in 37.8% and a shrunken liver in 15.3% (Supplementary Table S2). HIDA scan was diagnostic of EHBA in 80.0% of tested patients, and upper gastrointestinal endoscopy revealed varices in 90.9% (Supplementary Table S2). Overall in-hospital mortality was 20.4% (*n* = 20), with significant differences across aetiologies (χ²=12.47, *p* = 0.014); EHBA had the highest case-fatality rate (35.7%), followed by Wilson disease (16.7%) (Supplementary Table S3; Fig. 2A).

## Discussion

This study provides a comprehensive evaluation of the clinico-aetiological profile, determinants of decompensation and outcomes in paediatric CLD in a tertiary care setting in South India. Wilson disease emerged as the predominant aetiology (36.7%), followed by EHBA (28.6%), consistent with the distribution presented in Table 1 and Supplementary Table S3. This aligns with previous Indian studies reporting Wilson disease as a leading cause of CLD in older children [3, 6]. EHBA predominantly affected younger children, as reflected by the higher proportion in the < 1-year age group. The proportion of idiopathic cases (14.3%) highlights persistent diagnostic limitations, particularly the lack of access to comprehensive metabolic and genetic testing in resource-constrained settings [2]. Kayser–Fleischer rings were documented in 29.5% of the overall cohort; however, systematic sub-phenotyping of the Wilson disease subgroup (hepatic versus neurological versus mixed presentation) and treatment-compliance history were not consistently available from the retrospective records and could not be reliably reconstructed, which is acknowledged as a limitation and warrants prospective phenotyping. Treatment non-compliance was recorded as a precipitant of decompensation in 5 (6.3%) of decompensated children (Table 2), indicating that a proportion of episodes represented acute-on-chronic deterioration rather than de novo presentations; the retrospective records did not, however, permit reliable attribution of compliance status to individual aetiologies. The decompensation rate of 80.6% in our cohort reflects advanced disease at presentation (Table 2), likely due to delayed referral patterns typical of tertiary care centres [5]. Univariable analysis identified malnutrition, hyponatraemia, gastrointestinal bleeding at presentation, pneumonia and hepatic encephalopathy as factors significantly associated with decompensation (Table 3). Recurrent decompensation (≥ 2 episodes) was associated with higher odds of mortality on univariable analysis (OR 3.84, 95% CI 1.12–13.14, *p* = 0.032), underscoring the prognostic importance of episode frequency. Hyponatraemia was the most common precipitating factor (36.7%), followed by sepsis (35.4%), gastrointestinal bleeding (34.2%) and pneumonia (31.6%) (Table 3). These findings are consistent with the pathophysiological role of circulatory dysfunction and infection in precipitating acute decompensation in chronic liver disease [7, 8]. Portal hypertension was present in 37.7% of patients and endoscopic varices in 90.9% of those examined, consistent with reports in paediatric CLD [9], reinforcing the need for structured surveillance and timely management of variceal bleeding [10, 11]. 

Children with CLD may present with an acute liver failure (ALF) phenotype, in which the distinction from decompensated chronic disease is often blurred, particularly in autoimmune hepatitis and Wilson disease [12, 13]. In our cohort, hepatic encephalopathy was present in 29 children, of whom 65.5% had advanced (Grade III–IV) disease, including 12 (41.4%) with Grade IV hepatic coma (Supplementary Table S3); neurological manifestations including altered sleep–wake cycle (19.4%), seizures (13.2%) and flapping tremors (6.1%) were also documented across the cohort (Supplementary Table S1). This indicates that a substantial subset presented with severe hepatic dysfunction at the more acute end of the ALF–CLD spectrum. However, INR and prothrombin time were not uniformly recorded across the retrospective record set, and formal classification of ACLF or ALF using standardised coagulopathy thresholds was therefore not possible; this is acknowledged as a limitation. Prospective studies incorporating systematic coagulation profiling are required to characterise the ALF/ACLF burden in this population.

Malnutrition was highly prevalent (62.2% overall; 86.4% among children < 5 years) and was significantly associated with decompensation (OR 3.71, 95% CI 1.31–10.52, *p* = 0.013) (Table 3; Supplementary Table S3). This reflects the well-recognised bidirectional relationship between malnutrition and liver disease progression [8, 14]. Among patients with hepatic encephalopathy (*n* = 29), 65.5% had advanced disease (Grades III–IV), consistent with the severity distribution shown in Supplementary Table S3. Hepatic encephalopathy was significantly associated with decompensation (*p* < 0.001), reinforcing its role as a marker of severe hepatic dysfunction. These findings emphasise the need for early nutritional intervention and close monitoring for neurological complications.

Overall in-hospital mortality was 20.4%, with significant variation across aetiologies (χ²=12.47, *p* = 0.014) (Supplementary Table S3). EHBA had the highest case-fatality rate (35.7%), likely reflecting delayed presentation beyond the optimal window for Kasai portoenterostomy [15–17]. Mechanisms beyond surgical timing are also relevant: children with biliary atresia awaiting transplantation are at substantial risk of acute decompensation and ACLF-like deterioration, frequently triggered by infection [18]. Consistent with this, infective precipitants were common in our cohort (sepsis 35.4%, pneumonia 31.6%). Detailed surgical history (prior Kasai portoenterostomy and Kasai failure) and transplant-listing status were not consistently documented in the retrospective records, and liver transplantation was not readily accessible to most families in this setting; these gaps are acknowledged as limitations. Mortality in Wilson disease (16.7%) may be attributable to late diagnosis and advanced cirrhosis at presentation rather than lack of effective therapy [6, 19]. 

This study has limitations inherent to its retrospective single-centre design. As an admitted tertiary-referral cohort, the population over-represents severe and decompensated disease; the 80.6% decompensation rate is therefore not generalisable to the broader paediatric CLD population and reflects referral bias, delayed diagnosis and limited access to specialised care characteristic of low- and middle-income country settings. We report only in-hospital mortality and did not capture liver-transplantation referral, candidacy, waiting-list mortality or access to transplant services; given the severity of the cohort and the central role of transplantation in determining outcome, this is an important limitation. Incomplete metabolic evaluation contributed to the idiopathic category, and INR/prothrombin time, detailed Wilson disease phenotyping, EHBA surgical history and pneumococcal vaccination status were not consistently recorded, precluding more granular analyses. The absence of long-term follow-up precluded survival analysis. Prospective multicentre studies are needed to validate these findings.

## Conclusion

Paediatric CLD in this South Indian tertiary care setting presents predominantly as advanced disease with a high burden of decompensation (80.6%). Wilson disease and EHBA are the leading aetiologies. Malnutrition, hyponatraemia, gastrointestinal bleeding, pneumonia and hepatic encephalopathy are factors significantly associated with decompensation on univariable analysis. Aetiology-specific mortality differed significantly (*p* = 0.014), being highest in EHBA. Recurrent decompensation was associated with mortality on univariable analysis. Early diagnosis, nutritional optimisation, prompt infection management and structured electrolyte monitoring are critical to improving outcomes in this vulnerable population.

## Supplementary Information

Supplementary Material 1.

## Data Availability

The datasets generated and/or analysed during the current study are not publicly available because they contain potentially identifiable patient information, but anonymised data are available from the corresponding author (KJM) on reasonable request.

## Abbreviations

ACLF: Acute–on–chronic liver failure
AIH: Autoimmune hepatitis
ALF: Acute liver failure
BMI: Body mass index
CHF: Congenital hepatic fibrosis
CI: Confidence interval
CLD: Chronic liver disease
CMV: Cytomegalovirus
EHBA: Extrahepatic biliary atresia
GI: Gastrointestinal
HIDA: Hepatobiliary iminodiacetic acid
IAP: Indian Academy of Pediatrics
INR: International normalised ratio
OR: Odds ratio
PEM: Protein–energy malnutrition
PFIC: Progressive familial intrahepatic cholestasis
PT: Prothrombin time
SBP: Spontaneous bacterial peritonitis
SPSS: Statistical Package for the Social Sciences
TORCH: Toxoplasma, Other, Rubella, Cytomegalovirus, Herpes
USG: Ultrasonography
WHO: World Health Organization
