# Supplementary material for: Clinico-aetiological profile, risk factors for decompensation and outcomes in paediatric chronic liver disease: a retrospective cohort study from a tertiary care centre in South India
Source: BMC Pediatr. 2026 Aug 10;26:1129. doi: 10.1186/s12887-026-07499-7 (PMC13741526; doi:10.1186/s12887-026-07499-7)
Supplement: Supplementary file 1 [file 12887_2026_7499_MOESM1_ESM.docx]

# SUPPLEMENTARY MATERIAL

Manuscript: Clinico-aetiological profile, risk factors for decompensation and outcomes in paediatric chronic liver disease: a retrospective cohort study from a tertiary care centre in South India

## Supplementary Table S1. Clinical presentation: symptoms and signs (n=98)

| Feature | n | % of 98 |
| --- | --- | --- |
| **Symptoms** |  |  |
| Jaundice | 90 | 91.8 |
| Abdominal distension | 85 | 86.7 |
| High-coloured urine | 46 | 46.9 |
| Pale stools | 30 | 30.6 |
| GI bleeding | 28 | 28.6 |
| Pain abdomen | 22 | 22.4 |
| Altered sleep-wake cycle | 19 | 19.4 |
| Seizures | 13 | 13.2 |
| Pruritus | 12 | 12.3 |
| **Signs** |  |  |
| Icterus | 93 | 94.8 |
| Hepatomegaly | 82 | 83.7 |
| Ascites | 79 | 80.6 |
| Firm liver | 72 | 73.4 |
| Pallor | 69 | 70.4 |
| Splenomegaly | 65 | 66.3 |
| Pedal oedema | 39 | 39.8 |
| Kayser–Fleischer rings | 29 | 29.5 |
| Clubbing | 22 | 22.4 |
| Palmar erythema | 17 | 17.3 |
| Abdominal tenderness | 10 | 10.2 |
| Rickets | 7 | 7.1 |
| Flapping tremors | 6 | 6.1 |
| Cataract | 3 | 3.1 |

GI: gastrointestinal.

## Supplementary Table S2. Investigations and microbiological profile

| Investigation | Subgroup / Result | n tested | n positive (%) |
| --- | --- | --- | --- |
| Blood culture | Positive | 29 | 7 (24.1) |
|  | *Streptococcus pneumoniae* |  | 3 |
|  | *Klebsiella pneumoniae* |  | 2 |
|  | *Pseudomonas aeruginosa* |  | 1 |
|  | *Escherichia coli* |  | 1 |
| Urine culture | Positive | 12 | 6 (50.0) |
|  | *Escherichia coli* |  | 4 |
|  | *Enterobacter* species |  | 2 |
| USG abdomen | Coarse echotexture | 98 | 88 (89.8) |
|  | Portal hypertension |  | 37 (37.8) |
|  | Shrunken liver |  | 15 (15.3) |
| HIDA scan | Diagnostic of EHBA | 35 | 28 (80.0) |
| Liver biopsy | Diagnostic | 35 | 27 (77.1) |
| TORCH panel | CMV IgM positive | 45 | 24 (53.3) |
| Upper GI endoscopy | Varices present | 22 | 20 (90.9) |
| Autoimmune workup | AIH confirmed | 44 | 5 (11.4) |

USG: ultrasonography; HIDA: hepatobiliary iminodiacetic acid; EHBA: extrahepatic biliary atresia; GI: gastrointestinal; CMV: cytomegalovirus; AIH: autoimmune hepatitis; TORCH: Toxoplasma, Other, Rubella, CMV, Herpes.

## Supplementary Table S3. Complications, hepatic encephalopathy grading and outcomes (n=98)

| Variable | n | % (of relevant denominator) |
| --- | --- | --- |
| **Complications (n=98)** |  |  |
| Malnutrition | 61 | 62.2 |
| Portal hypertension | 37 | 37.7 |
| Hepatic encephalopathy | 29 | 29.6 |
| Variceal bleeding | 20 | 20.4 |
| Spontaneous bacterial peritonitis | 5 | 5.1 |
| **HE grading (n=29)** |  |  |
| Grade I (mild) | 6 | 20.7 |
| Grade II (moderate) | 4 | 13.8 |
| Grade III (severe) | 7 | 24.1 |
| Grade IV (coma) | 12 | 41.4 |
| **Outcomes** |  |  |
| Survived | 78 | 79.6 |
| Died | 20 | 20.4 |
| **Aetiology-specific mortality (n=20 deaths) — χ²=12.47, p=0.014** |  |  |
| EHBA (n=28) | 10 | 35.7 |
| Wilson disease (n=36) | 6 | 16.7 |
| Idiopathic CLD (n=14) | 3 | 21.4 |
| Metabolic liver disease (tyrosinaemia) (n=4) | 1 | 25.0 |
| Others (n=16) | 0 | 0.0 |

HE: hepatic encephalopathy; EHBA: extrahepatic biliary atresia; CLD: chronic liver disease. For the test of aetiology-specific mortality, aetiologies were grouped into five categories to ensure adequate expected cell counts: EHBA, Wilson disease, idiopathic CLD, metabolic liver disease (tyrosinaemia) and "Others"; the "Others" category (n=16) comprises Budd–Chiari syndrome (n=7), autoimmune hepatitis (n=5) and other aetiologies (n=4), corresponding to the categories in Table 1 of the main manuscript. Aetiology-specific mortality was tested using the chi-square test (χ²=12.47, df=4, p=0.014).
